# Cerebrospinal fluid C-reactive protein to diagnose healthcare-associated meningitis after neurosurgical procedures: An exploratory retrospective diagnostic accuracy study

**DOI:** 10.1016/j.bas.2026.107466

**Published:** 2026-10-07

**Authors:** M. Foppen, I.O. Kommers, S.E. Olie, H.H.M. Schotman, D. Verbaan, M.C. Brouwer

**Affiliations:** a Department of Neurosurgery, Amsterdam Neuroscience, Amsterdam UMC, University of Amsterdam, the Netherlands; b Department of Neurosurgery, Erasmus Medical Center, Erasmus University Rotterdam, the Netherlands; c Department of Neurology, Amsterdam Neuroscience, Amsterdam UMC, University of Amsterdam, the Netherlands; d Department of Laboratory Medicine, Amsterdam UMC, Vrije Universiteit Amsterdam, the Netherlands

**Keywords:** Humans, C-Reactive protein, Cerebrospinal fluid, Meningitis, Neurosurgery, Neurosurgical procedures

## Abstract

**Introduction:**

Neurosurgical procedures are frequently complicated by postoperative meningitis. Current diagnostic methods are insufficient and time-consuming. Cerebrospinal fluid C-reactive protein (CSF CRP) has shown excellent diagnostic accuracy for patients with a community-acquired meningitis and can be determined quickly.

**Research question:**

This study investigates the diagnostic accuracy of CSF CRP for detecting postoperative meningitis.

**Materials and methods:**

We performed a retrospective monocenter cohort study, including patients in whom a postoperative meningitis was suspected within 30 days after neurosurgery. To assess the diagnostic accuracy of CSF CRP, the sensitivity, specificity, positive predictive value (PPV), negative predictive value (NPV), receiver operating curve (ROC) and area under the curve (AUC) with 95% confidence interval (CI) were calculated.

**Results:**

Forty-five patients were included of whom 8 (18%) were diagnosed with postoperative meningitis. Two of eight patients (25%) with a meningitis had a detectable CSF CRP concentration (>0.30 mg/L) compared to eighteen of 37 (49%) without a meningitis. With a cut-off value of 0.30 mg/L, CSF CRP had a 25% sensitivity, 51% specificity, 10% PPV and 76% NPV. The AUC was 0.43 (95% CI 0.22-0.66).

**Discussion and conclusion:**

The diagnostic accuracy of CSF CRP to diagnose a postoperative meningitis is poor and treatment decisions should not be based on this parameter.

## Highlights

• The diagnostic accuracy of CSF CRP to diagnose postoperative meningitis is poor.
• The decision to start or commence antibiotics should not be based on CSF CRP.
• Larger studies are required to determine if CSF CRP may be helpful in subgroups.

## 1 Introduction

Postoperative bacterial meningitis is a common complication after neurosurgical procedures and occurs in approximately 2-25% of all cases (Jamjoom et al., 2018; Lozier et al., 2002; Dorresteijn et al., 2019; Korinek et al., 2008; Reichert et al., 2002). Most common symptoms are fever, headache, photophobia, neck stiffness, nausea or a decreased level of consciousness, but due to the primary neurosurgical condition clinical characteristics may be difficult to assess. Postoperative meningitis is potentially life-threatening and must be treated with antibiotics as soon as possible. It is diagnosed based on the patient's clinical status combined with the results of cerebrospinal fluid (CSF) cultures (gold standard), and inflammatory CSF and serum characteristics (total CSF leukocyte, decreased glucose level and total protein level in the CSF, serum C-reactive protein (CRP) and leukocyte count). However, a major limitation of this method is that, while CSF leukocyte count, glucose and protein are quickly available, results are often unreliable in patients after neurosurgical procedures, and definitive results from CSF cultures may take up to a week. Therefore, it is good clinical practice to start with antibiotics while awaiting the culture results, when a postoperative meningitis is suspected. Nevertheless, the majority of patients presenting with clinical features suggestive of meningitis are found not to have meningitis upon further evaluation. This leads to inappropriate use of antibiotics and a prolonged hospital stay (Hoogmoed et al., 2017).

In order to diagnose postoperative meningitis more accurately and to reduce the inappropriate use of antibiotics, other biomarkers than currently used in clinical practice are required. The concentration of cerebrospinal fluid CRP could serve as such a marker. A major advantage of CSF CRP is that it is readily available and may be associated with inflammation, similar to serum CRP (sCRP). A recent study reported that the concentration of CSF CRP is an excellent biomarker to predict bacterial meningitis in non-neurosurgical patients in whom a community-acquired meningitis was suspected (Olie et al., 2025a). CSF CRP alone had an AUC of 0.99 (95% CI: 0.97–1.00) with a sensitivity 100%. This study however, excluded all patients with a recent neurosurgical operation or neurosurgical devices in situ. Factors such as iatrogenic disruption of the blood brain barrier, the expected increase of postoperative sCRP and the placement of foreign objects (e.g. ventricular catheters and aneurysm clips or coils) inside the central nervous system could alter the CSF CRP concentration (Fountas et al., 2009; Wang et al., 2025). Therefore, it is not possible to extrapolate the results of the aforementioned study to a neurosurgical population. In June 2024 CSF CRP became available as routine measurement in our institution and is available within 1 h of arrival in the laboratory. Following publication of the implementation study of CSF CRP in community-acquired bacterial meningitis in April 2025 we decided to include CSF CRP as routine measurement in neurosurgical patients suspected of a central nervous system (CNS) infection.

The aim of this study is to evaluate the diagnostic accuracy of CSF CRP to diagnose a postoperative meningitis in a neurosurgical population.

## 2 Methods

This study is a retrospective, single center cohort study, performed in the Amsterdam UMC from the 1st of April 2025 until the 15th of December, 2025. Patients were included if they 1) were above 18 years, 2) underwent any form of cranial or spinal neurosurgical procedure 3) had a clinical suspicion of a postoperative meningitis within the first 30 days after surgery and cerebrospinal fluid was drawn to evaluate CSF characteristics including CRP. A meningitis was suspected if patients exhibited fever (>38°C) and any of the following symptoms during admission: headache, neck stiffness, photophobia, nausea, vomiting or no identifiable alternative source of the fever.

Patients were excluded if 1) the indication for surgery was related to a central nervous system infection, such as an external ventricular drain (EVD) for a bacterial meningitis, evacuation of an intracranial, intraparenchymal abscess or a subdural empyema, 2) they were already treated with antibiotics for meningitis, 3) no CSF CRP could be determined.

CSF could be collected in multiple ways: 1) from EVD catheters by catheter port sampling, 2) from internal ventricular shunts by tapping the shunt reservoir, or 3) through lumbar puncture. Since routine CSF sampling is associated with a higher risk of ventricular catheter associated infections in patients with ventricular drains, our institutional protocol warrants that CSF should only be obtained when clinically indicated and before antibiotic treatment is started. Antibiotics were started if the CSF (i.e. increased leukocyte count and leukocyte/erythrocyte ratio) was suspected for a CNS infection or if it was inconclusive but there was a high clinical suspicion of meningitis, awaiting the definitive results of the CSF cultures. Our institutional protocol states that when a ventricular catheter associated meningitis is suspected, intravenous vancomycin and ceftazidime are started and when a non-ventricular catheter related postoperative meningitis is suspected, intravenous flucloxacillin and ceftazidime are started.

In some patients CSF was sampled more than once during the 30 day period after surgery because a postoperative meningitis was suspected at multiple moments. To ensure a reliable assessment of CSF CRP, only the results of CSF characteristics and CSF cultures from the first drawn CSF (before start of antibiotics) were included in this analysis. In our facility, it is standard practice to determine CSF CRP, leukocytes, erythrocytes and CSF cultures when a postoperative meningitis is suspected. Ethical approval is not required in the Netherlands for a retrospective study with anonymized patient data such as our study (Ten Dam et al., 2022).

### 2.1 Perioperative antimicrobial measures

According to our institutional protocol, all patients received 2 g of cefazolin intravenously preoperatively as antibiotic prophylaxis for neurosurgical procedures. All surgeries were performed in the operating theatre under general anesthesia and under sterile conditions. All ventricular shunts (external or permanent) placed in this study are made of a plain silicone catheters, not impregnated with antibiotics. Patients admitted to the intensive care unit were additionally treated with prophylactic selective bowel decontamination, which consisted of 3 g cefotaxime daily for maximum of four days.

### 2.2 Data collection and outcomes

The following variables were retrieved from the patients' electronic medical file: age, sex, indication for surgery, type of surgery, duration of EVD placement in days, concentration of serum CRP, CSF CRP, CSF leukocyte and erythrocyte count and the results from CSF cultures. The index test of the study was the CRP concentration measured in CSF using the Roche Diagnostics (Almere, the Netherlands) CRP4 Tina-quant C-reactive Protein IV assay, according to manufacturer's instructions. The limit of detection (LoD) of the assay was 0.30 mg/L, which we predefined to be the cut-off value for a positive test, based on the results in community acquired bacterial meningitis patients (Olie et al., 2025a). All patients with a CRP value below the limit of detection were assigned the value 0.00 mg/L. A postoperative meningitis was defined according to the criteria of the Infectious Diseases Society of America (IDSA) for healthcare-associated ventriculitis and meningitis; 1) organism cultured from CSF, and 2) at least two of the following symptoms: fever >38°C or headache, meningeal signs, or cranial nerve signs (Tunkel et al., 2017). A sensitivity analysis was performed with all patients in whom the IDSA criteria were not fulfilled but a clinical suspicion of postoperative meningitis remained present. Such cases were scored independently by two authors (MF and SEO) as having a clinical suspected health-care associated CNS infection or no CNS infection (which included culture of contaminants in the CSF). If there was disagreement between adjudicators, a specialist in neurological infections (MCB) was consulted to make a final diagnosis.

### 2.3 Statistical analysis

The normality of continuous variables was assessed with the Shapiro-Wilk test and considered normally distributed with a W-value > 0.9. A mean and standard deviation (SD) were calculated for normally distributed variables. A median and interquartile range (IQR) was calculated for not normally distributed variables. To determine the diagnostic accuracy of CSF CRP the sensitivity, specificity, positive predictive value (PPV), negative predictive value (NPV), the receiver operating curve (ROC) and the area under the curve (AUC) with 95% CI were determined. The ROC and AUC with 95% CI were also determined for the total CSF leukocyte count and the total CSF leukocyte count combined with the CSF CRP concentration (by including both parameters in a logistic regression model). To calculate the sensitivity, specificity, PPV and NPV the concentration of CSF CRP was cut-off at the limit of detection threshold (0.30 mg/L). Additionally, we determined the Youden's index to evaluate which cut-off had the optimal combination of sensitivity and specificity. The Pearson correlation test (r-value with 95% CI) was calculated to evaluate the association of the CSF CRP concentration with total CSF leukocyte and CSF erythrocyte count. The correlation coefficient (r-value) was considered as follows: 0.00-0.09 negligible, 0.10-0.39 weak, 0.40-0.69 moderate, 0.70-0.89 great, 0.90-1.00 very strong (Schober et al., 2018).

This study is an exploratory diagnostic accuracy study and no preliminary data regarding the performance characteristics of the index test were available, and as such a sample size calculation could not be performed. All statistical analyses were performed using IBM SPSS statistics, version 28.0 and R version 4.3.4 (R Foundation for Statistical Computing, Vienna, Austria; http://www.R-project.org/) (Team, 2020). This study adhered to the Standards for Reporting Diagnostic accuracy studies (STARD) checklist.

## 3 Results

A total of 45 patients were included (see Fig. 1) with a mean age of 52 (SD 18) years and most patients, 27 of 45 (60%), were male (see Table 1). The indications for neurosurgery were subarachnoid hemorrhage with concomitant hydrocephalus in 14 (31%), followed by resection of a tumor in 10 (22%) and traumatic brain injury in 10 (22%). The most common performed type of surgery was placement of an EVD in 22 (49%), followed by resection of a tumor in 8 (18%) and a craniectomy in 5 (11%). The median period between surgery and the moment of CSF sampling was 7 days (IQR 4-10). The median concentration of CSF CRP in the whole cohort was 0.00 mg/L (IQR 0.00-0.41) and in 20 (44%) patients the CSF CRP was above the detection limit. Eight patients (18%) developed a postoperative meningitis in the first 30 days after surgery fulfilling the IDSA criteria for healthcare associated-meningitis/ventriculitis (Table 1 and Fig. 2). The most common causative microorganism was *Cutibacterium acnes* (2/8, 25%, Table 2). In 6 additional patients (13%) the authors scored the patient as having culture negative postoperative meningitis (Supplementary Fig. 5 and Table 1).

**Fig. 1:**
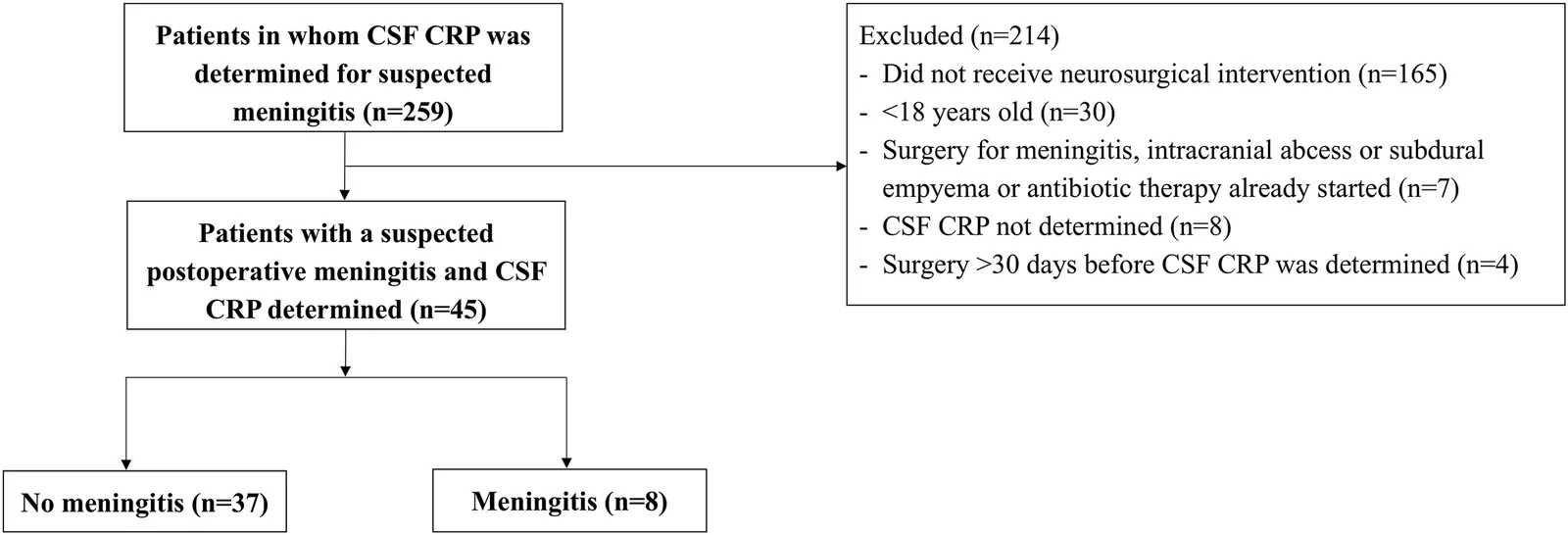
Flowchart of patient selection. The study cohort consisted of 45 patients who were selected from 259 patients who presented to our facility with the suspicion of a meningitis, nosocomial or postoperative, during the study period. In eight patients CSF CRP could not be determined due to hemolysis of the CSF before it could be analyzed.

**Table 1:** EVD, external ventricular drain; ICP, intracranial pressure monitor; LPD, lumboperitoneal drain; IQR, interquartile range; VPD, ventricular peritoneal drain; SD, standard deviation.

Variable	Total (n = 45)	No meningitis (37)	Meningitis (n = 8)
**Demographic variables**			
Age, mean (SD)	52 (18)	54 (18)	43 (18)
Male, n (%)	27 (60)	23 (63)	4 (50)
**Indication for surgery**			
Subarachnoid hemorrhage, n (%)	14 (31)	13 (35)	1 (13)
Intraparenchymal hemorrhage, n (%)	2 (4)	1 (3)	1 (13)
Tumor, n (%)	10 (22)	7 (19)	3 (38)
Cerebellar hemorrhage, n (%)	2 (4)	1 (3)	1 (13)
Cerebellar infarction, n (%)	2 (4)	2 (5)	0 (0)
Traumatic brain injury, n (%)	10 (22)	10 (27)	0 (0)
Other, n (%)	5 (11)	3 (8)	2 (25)
**Type of surgery**			
EVD + coiling, n (%)	12 (27)	11 (30)	1 (13)
EVD, n (%)	11 (25)	10 (27)	1 (13)
Tumor resection, n (%)	8 (18)	6 (16)	2 (25)
Craniectomy + EVD/ICP monitor, n (%)	5 (11)	5 (14)	0 (0)
VPD/LPD placement or revision, n (%)	4 (9)	2 (5)	2 (25)
Posterior fossa decompression + EVD, n (%)	3 (7)	1 (3)	1 (13)
Other¥	2 (4)	1 (3)	1 (13)
Duration EVD, days (SD)	15 (10)	15 (11)	13 (5)
**CSF and serum chemistry**			
Days from surgery to CSF sampling, median (IQR)	7 (4-10)	7 (4-10)	9 (5-11)
CSF CRP mg/L, median (IQR)	0 (0-0.41)	0 (0-0.41)	0 (0-0.37)
CSF CRP >0.30 mg/L, n (%)	20 (44)	18 (49)	2 (25)
CSF leukocyte count^a^, median (IQR)	201 (46-628)	215 (52-561)	134 (15-1830)
CSF erythrocyte count^a^, median (IQR)	9500 (600-45950)	11100 (675-48250)	3900 (375-42275)
Serum CRP mg/L, median (IQR)^b^	19 (6-108)	22 (14-101)	47 (10-90)

¥ Other types of surgery were an endoscopic third ventriculostomy and a craniotomy.

a 10^6/L.

b Serum CRP was known for 26 patients.

**Fig. 2:**
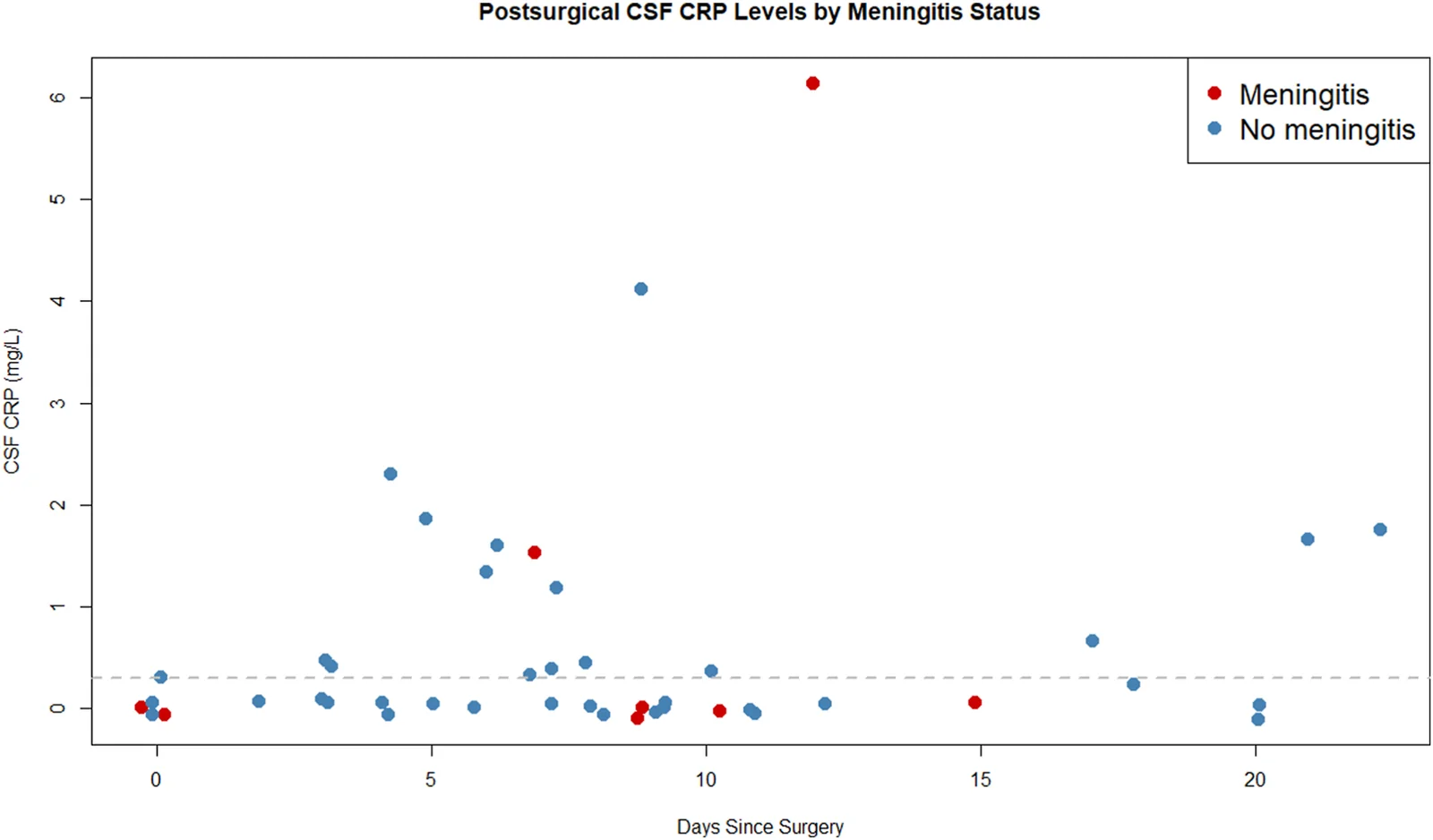
The dotted grey line indicates a CSF CRP concentration of 0.30 mg/L. The red dots represent the CSF CRP concentration of patients who developed a healthcare-associated postoperative meningitis (according to the IDSA criteria). The blue dots represent the CSF CRP concentrations of patients who did not develop a postoperative meningitis.

**Table 2:** Cultured microorganisms in the CSF of patients with a meningitis. In one patient more than one microorganism was cultured.

Cultured microorganisms in CSF	n (%)
*Cutibacterium acnes*	2 (22%)
*Acinetobacter*	1 (11%)
*Serratia marcescens*	1 (11%)
*Stafylococcus aureus*	1 (11%)
*Stafylococcus epidermidis*	1 (11%)
*Stafylococcus hemolyticus*	1 (11%)
*S. hemolyticus, S. epidermidis, C. acnes*	1 (11%)

Two of 8 patients (25%) with postoperative meningitis fulfilling the IDSA criteria had a detectable concentration of CSF CRP (>0.30 mg/L) and eighteen of 37 (49%) patients without a meningitis had a CSF CRP concentration >0.30 mg/L (Fig. 1). When the predefined CRP concentration cut-off of 0.30 mg/L was used, the sensitivity of CSF CRP was 25% and the specificity was 51%. The PPV was 10% and the NPV was 76%. The AUC of CSF CRP was 0.43 (95% CI 0.22-0.66), (Fig. 3).

**Fig. 3:**
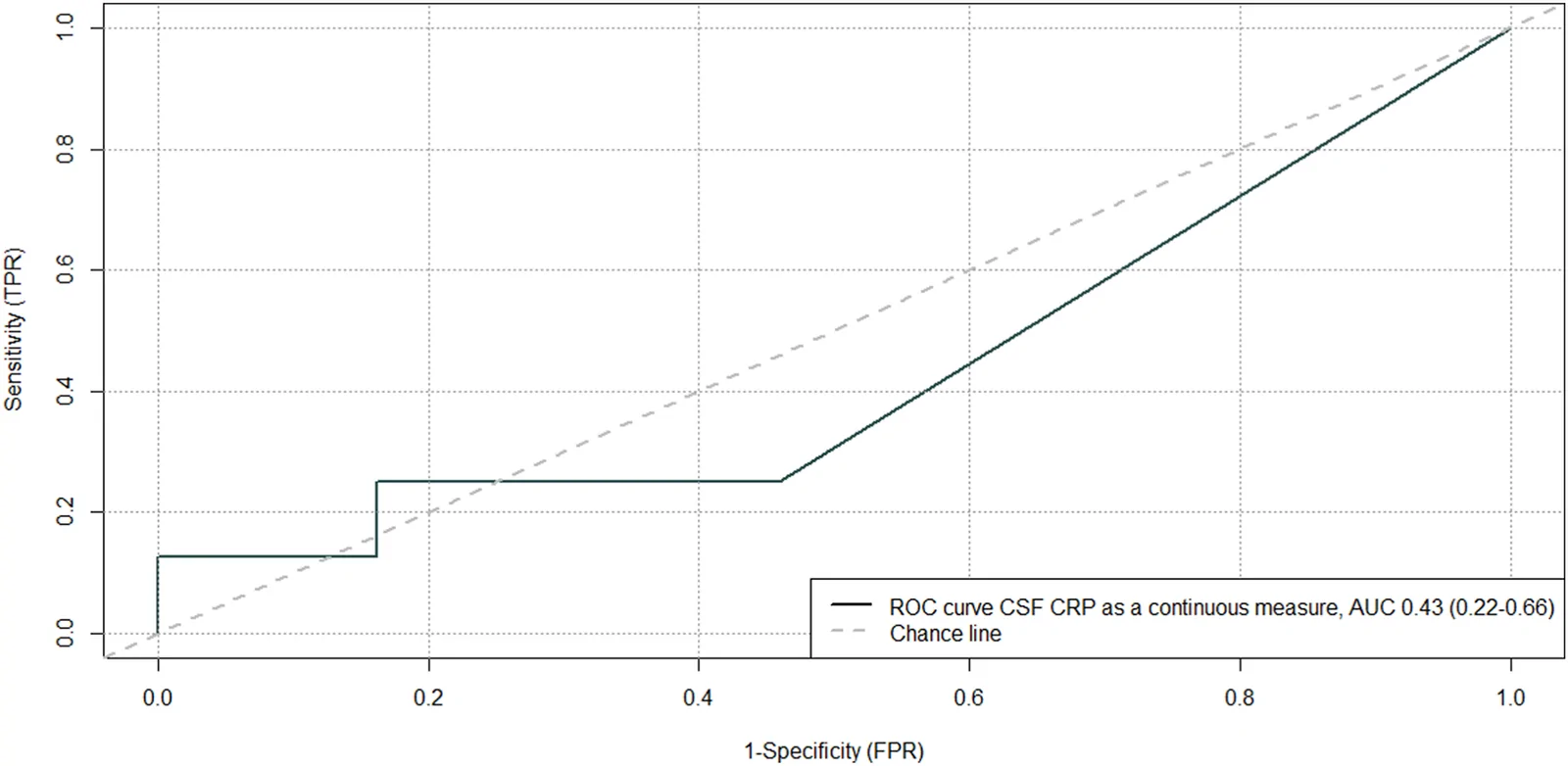
The ROC curve of the CSF CRP concentration with AUC and 95% CI.

The optimal CSF CRP concentration cut-off value was determined at 1.36 mg/L or higher (Youden's index). For this value the sensitivity remained 25% but the specificity increased to 84%.

The AUC of the CSF leukocyte count was 0.49 (95% CI 0.28-0.71) (Supplemental Fig. 1a). Combining CSF leukocyte count and CSF CRP did not result in a better diagnostic accuracy (AUC 0.48 [95% CI 0.26-0.70], Supplemental Fig. 1b).

The CSF CRP concentration was moderately correlated with the total CSF leukocyte count (r = 0.63, 95% CI 0.42-0.79, p < 0.001), the correlation with the total CSF erythrocyte count was negligible (r = 0.04, 95% CI -0.26-0.34, p = 0.78) and weakly correlated with the serum CRP concentration (r = 0.37, 95% CI -0.03-0.66, p = 0.07) (Supplemental Fig. 2a–c).

In the sensitivity analysis including both clinical suspected and microbiologically confirmed postoperative meningitis thirteen (29%) patients were included (Supplemental Table 1). Six of these 13 patients (46%) with a meningitis had a detectable concentration of CSF CRP (>0.30 mg/L) and thirteen of 32 (41%) patients without a meningitis had a detectable CSF CRP concentration (see Supplemental Fig. 3). The CSF CRP concentration with cut-off of 0.30 mg/L, had a 46% sensitivity, 59% specificity, 35% PPV, 76% NPV. The AUC was 0.59 (95% CI 0.41-0.78) (Supplementary Fig. 4). The optimal CSF CRP concentration cut-off in the sensitivity analysis was determined at 1.22 mg/L or higher (Youden's index). For this value the sensitivity remained 46% but the specificity increased to 91%.

## 4 Discussion

In this explorative retrospective monocenter cohort study, the diagnostic accuracy of the CSF CRP concentration to predict a postoperative meningitis was poor. Therefore, CSF CRP concentrations should not guide clinicians in the decision to commence or withhold antibiotic therapy for neurosurgical patients with suspected postoperative meningitis.

The diagnostic accuracy of CSF CRP concentration and whether it could possibly predict or rule out a postoperative meningitis when clinically suspected has not been studied previously in clinical practice. A postoperative meningitis occurs in approximately 2-25% of patients after neurosurgical procedures and could be lethal if untreated. Therefore, there is a low threshold to start antibiotics while awaiting CSF culture results. Therefore, the most important clinical objective of this study was to investigate whether CSF CRP could rule out a meningitis (high sensitivity), enabling physicians to safely withhold antibiotic therapy. A diagnostic test with high specificity for predicting a postoperative meningitis would offer limited clinical benefit, as empirical antibiotic therapy is started irrespective of the test findings. The CRP found in the CSF is produced in the liver and leaks through the blood-brain-barrier (BBB). Neurosurgical procedures disrupt the BBB and studies have shown that neurosurgical procedures also increase the concentration of serum CRP in the postoperative period (Mirzayan et al., 2007). Therefore, it is understandable that almost half of the patients without a meningitis had CSF CRP concentrations above the detectable limit (0.30 mg/L). This is particularly relevant given that the serum CRP levels in our cohort were indeed elevated when a postoperative meningitis was suspected. An additional explanation for elevated CSF CRP levels in patients without meningitis is that bleeding during surgery also leads to CRP leakage towards the cerebrospinal fluid.

Furthermore, it is noteworthy that 75% of the patients in the meningitis group, had a non-detectable concentration of CSF CRP. Especially since all patients with a community-acquired meningitis in a clinical implementation cohort were found to have an elevated CSF CRP concentration (Olie et al., 2025a). In healthcare-associated meningitis it is likely that the infection is confined to the central nervous system, and no or only a very limited systemic inflammatory response is present. Therefore, other inflammatory markers of infection that are produced locally by leukocytes, such as interleukin-6 (IL6), heparin binding protein (HBP) or neutrophil gelatinase-associated lipocalin (NGAL), may have a higher diagnostic yield (Lin et al., 2024; Olie et al., 2024, 2025b). The surgical indications and procedures for patients with a non-detectable concentration of CSF CRP and a postoperative meningitis varied: including EVD placement for SAH associated hydrocephalus, EVD placement for intraparenchymal hemorrhage associated hydrocephalus, Ommaya reservoir placement and ventriculostomy for an obstructive hydrocephalus, ventricular peritoneal drain revision and glioblastoma resection combined with EVD placement due to a postoperative intraventricular hemorrhage. Thus, the type of surgical procedure or its indication did not appear to have a significant impact. However, the limited sample size in our study prevents us from drawing definitive conclusions on this matter.

Several studies have described the CSF CRP concentration in the postoperative period after neurosurgical procedures. In a study of 236 patients who were operated for intracranial aneurysms, the per-operatively collected CSF showed higher levels of CRP in those who would later develop meningitis (Guo et al., 2021). This study however, did not describe the diagnostic value of CSF CRP at the moment meningitis was suspected.

Another study in 41 patients with aneurysmal SAH, treated surgically or endovascular, measured CSF CRP after 0, 1, 2, 3, 5, 7 and 9 days (Fountas et al., 2009). The CSF CRP concentration generally peaked approximately three days after the ictus. The diagnostic accuracy of CSF CRP for postoperative meningitis was not described. A similar course of postoperative CSF CRP was found in another study by including 103 patients with traumatic brain injury (TBI) (Wang et al., 2025). The CSF CRP concentration spiked three days after the initial trauma, and higher levels were associated with a more severe form of TBI and worse outcome. Again no analysis was done to determine the diagnostic value for detecting postoperative meningitis. This observed increased concentration of CSF CRP after neurosurgery and neurotrauma explains the high proportion of patients without meningitis in our cohort who had an elevated CSF CRP.

### 4.1 Limitations and future considerations

Our study has several limitations. First, the sample size of this study was relatively small. As there were no previous studies addressing this topic we performed a pilot study to enable a power calculation to determine an appropriate sample size for future studies. However, given the poor performance of CSF CRP, it is unlikely that further larger cohort studies will detect a meaningful effect. Another consequence of the modest sample size was the inability to assess CSF CRP concentrations within individual subgroups, such as patients with SAH, TBI, intracranial tumors or VPD related surgery. It is conceivable that CSF CRP concentrations vary between patient cohorts and that CSF CRP could nonetheless retain some degree of clinical utility.

This study did not assess serum leukocyte count, nor did it evaluate other CSF indicators of inflammation such as interleukin-6 (IL-6) or procalcitonin. In our facility serum leukocytes are not determined routinely when a postoperative meningitis is suspected because these are often elevated during the postoperative period, independent of infection status. It is also not standard practice to determine CSF IL-6 or procalcitonin levels. Some studies report a better diagnostic value of CSF IL6 than CSF CRP, which may therefore be a possible target for future studies predicting postoperative meningitis (Lenski et al., 2019). A recent systematic review demonstrated that the available literature regarding these biomarkers is scarce and that the quality of evidence is low (Dorresteijn et al., 2019). Large multi-center prospective diagnostic accuracy studies on neurosurgical patients with suspected postoperative meningitis are needed to determine the value of novel biomarkers in CSF.

## 5 Conclusion

The diagnostic accuracy of CSF CRP to predict a postoperative meningitis after neurosurgical procedures is poor. Physicians should not base their decision to commence or withhold antibiotics on the CSF CRP concentration when a postoperative meningitis is suspected. Future studies focused on subgroups are required to determine whether CSF CRP concentrations offer additional value for specific patients. However, based on our results it may be more prudent to study other potential markers of healthcare associated meningitis, such as IL6, HBP or NGAL.

## Ethics declaration

The authors have NOT obtained informed consent from participants or their legal representatives. Ethical approval is not required in the Netherlands for a retrospective study with anonymized patient data such as our study (non WMO).

This study was performed in compliance with relevant laws, regulatory frameworks and guidelines where the research took place. Ethics committee approval was not required under relevant laws and institutional guidelines. Ethical approval is not required in the Netherlands for a retrospective study with anonymized patient data such as our study.

## Declaration of competing interest

The authors declare that they have no known competing financial interests or personal relationships that could have appeared to influence the work reported in this paper.
