# Supplementary material for: Cerebrospinal fluid C-reactive protein to diagnose healthcare-associated meningitis after neurosurgical procedures: An exploratory retrospective diagnostic accuracy study
Source: Brain Spine. 2026 Oct 7;6:107466. doi: 10.1016/j.bas.2026.107466 (PMC13677407; doi:10.1016/j.bas.2026.107466)
Supplement: Supplementary file 1 [file mmc1.docx]

**Supplemental figure 1a**

*The ROC curve of CSF leukocyte count*

**Supplemental figure 1b**

*The ROC curve of CSF leukocyte count combined with CSF CRP.*

**Supplemental figure 2a**

**Supplemental figure 2b**

**Supplemental figure 2c**

**Supplemental figure 3**

*The dotted grey line indicates a CSF CRP concentration of 0.30mg/L. The red dots represent the CSF CRP concentration of patients who developed a culture negative healthcare-associated postoperative meningitis in the sensitivity analysis (n=13). The blue dots represent the CSF CRP concentrations of patients who did not develop a postoperative meningitis.*

**Supplemental figure 4**

*The ROC curve of the CSF CRP concentration with AUC and 95% CI in the sensitivity analysis.*

**Supplementary Table 1**

| **Case#** | **IDSA criteria** | **Sensitivity analysis** | **Description** |
| --- | --- | --- | --- |
| 1 | No meningitis | Meningitis | Headache, altered behavior and confusion after tumor resection. CSF 227 leukocytes, 8000 erythrocytes, CSF CRP <0.3, negative CSF culture. Other postoperative complications ruled out by imaging. Recovered after one and a half week of antibiotic (AB) treatment. |
| 2 | No meningitis | Meningitis | Fever, headache and neck stiffness after tumor resection. CSF 5300 leukocytes, 166.000 erythrocytes, CSF CRP 2.27, negative CSF culture. Other postoperative complications ruled out by imaging. Recovered after two weeks of AB treatment. |
| 3 | No meningitis | Meningitis | Fever, CSF leakage after EVD removal for subarachnoid hemorrhage (SAH), increased leukocyte count (1366), low erythrocyte count (0) negative CSF culture. Other postoperative causes ruled out by imaging. Good recovery after two weeks of AB treatment. |
| 4 | No meningitis | Meningitis | Decreased GCS, somnolence and aphasia after SAH and EVD placement. Leukocyte count 2336 in CSF, no microorganism cultured. A few days later developed the patient a fever and multiple microorganisms cultured in CSF. |
| 5 | No meningitis | Meningitis | Six days after traumatic brain injury with an EVD in situ developed patient a fever, leukocyte count 3621 in CSF, CSF culture negative. Ten day AB treatment. |
| 6 | No meningitis | Meningitis | Infected autologous material after intramedullary tumor resection, complicated by postoperative bleeding. Initial CSF cultures negative but treated multiple times with AB in meningitis dosage and during surgical exploration to remove infected material an abscess was found. |
| 7 | Meningitis | No meningitis | VPD visible through skin with CSF leakage, no complaints of meningitis CSF. Initial CSF culture positive for C. acnes, CSF leukocyte count 12. Cultures afterwards all negative. C. acnes was considered to be contamination. |
| *All cases which had a different outcome after clinical consideration. Leukocyte and erythrocyte count *10^6.* | | | |
